# Mass cytometry analysis reveals attrition of naïve and anergized self-reactive non-malignant B cells in chronic lymphocytic leukemia patients

**DOI:** 10.3389/fonc.2022.1020740

**Published:** 2022-10-31

**Authors:** Thibault Andrieu, Paul Mondière, Pierre-Emmanuel Jouve, Sébastien Dussurgey, Victor Malassigné, Hugo Servanton, Lucille Baseggio, Frédéric Davi, Anne-Sophie Michallet, Thierry Defrance

**Affiliations:** ^1^ Centre de Recherche en Cancérologie de Lyon, INSERM U1052, CNRS UMR5286, Université de Lyon, Université Lyon 1, Centre Léon Bérard, Lyon, France; ^2^ CIRI Centre International de Recherche en Infectiologie, Univ Lyon, Université Claude Bernard Lyon 1, Inserm, U1111, CNRS, UMR5308, ENS Lyon, Lyon, France; ^3^ AltraBio SAS, Lyon, France; ^4^ SFR Biosciences (UAR3444/CNRS, US8/INSERM, ENS de Lyon, UCBL) AniRA-cytometry, Lyon, France; ^5^ France Biotech, Paris, France; ^6^ Department of Hematology, Hôpital Lyon Sud, Hospices Civils de Lyon, Pierre Bénite, France; ^7^ Department of Hematology, APHP, Hôpital Pitié-Salpêtrière and Sorbonne University, Paris, France; ^8^ Department of Hematology and Medical Oncology, Centre Léon Bérard, Lyon, France

**Keywords:** chronic lymphocytic leukemia, CLL, B cells, deep phenotyping, mass cytometry

## Abstract

Chronic Lymphocytic Leukemia (CLL) is characterized by the progressive accumulation of monoclonal mature B lymphocytes. Autoimmune complications are common in CLL occurring in up to a quarter of all patients during the course of the illness. Etiology of autoimmunity in CLL is unknown but it is widely admitted that the pathogenic auto-Abs do not originate from the tumoral clone but from the non-malignant B cell pool. This indicates that the developmental scheme of non-malignant B cells could also be perturbed in CLL patients. To address this question, we have designed a B cell-centered antibody panel and used time-of-flight mass cytometry to compare the residual non-malignant B cell pool of CLL patients with the peripheral B cell pool of age-matched healthy donors. We show that the non-malignant B cell compartment of the patients is characterized by profound attrition of naïve B cells and of a population of anergized autoreactive B cells, suggesting impaired B cell lymphopoeisis as well as perturbations of the B cell tolerance checkpoints.

## Introduction

Chronic lymphocytic leukemia (CLL) is a B cell malignancy characterized by progressive accumulation of monoclonal mature B lymphocytes in primary and secondary lymphoid tissues. Although CLL is a relatively slow-progressing disease, the overall patients prognosis also heavily depends on immune alterations associated with CLL. It is well accepted that a global T cell defect is associated with the disease, encompassing an impairment of T_FH_ (1–4) and cytotoxic T cell functions (5, 6), skewing towards Tregs differentiation (1, 7) and functional exhaustion (8). By contrast, alterations of the non-malignant B cell compartment of CLL patients and their possible contribution to the immune dysfunctions associated with this disease have been poorly documented. Still, there are clues in the literature suggesting that this is indeed the case. It is a well-known fact that up to a quarter of CLL patients present autoimmune manifestations (9) characterized by production of autoreactive antibodies (Abs) that primarily target cells of the hematopoietic lineage: red cells, platelets or granulocytes. As the pathogenic auto-Abs do not originate from the tumoral clone but rather from the non-malignant B cell pool (10, 11), it is probable that the tumor also leads to functional or developmental anomalies of the residual healthy B cell compartment of the patients.

The scarcity of information available on composition and functionality of the healthy B cell pool of CLL patients is possibly due to two technical bottlenecks that complicate its analysis. First, the low abundance of circulating healthy B cells in CLL weighs heavily on dissection of this compartment especially when it comes to analysis of rare B cell subtypes that can represent less than 1% of the healthy B cell pool. Second, phenotypical discrimination of healthy and leukemic B cells is not a straightforward matter as certain CLL markers such as CD5 are also expressed on certain healthy B cell subsets.

Here we report the results of a pilot study in which we carried out an unprecedented in-depth phenotypical analysis of the healthy peripheral B cell pool of treatment-naïve CLL patients using mass cytometry. We show that, despite its strong size reduction, the sparse non-malignant B cell compartment of CLL can be interrogated and analysed using high-dimensional phenotypic characterization. We also show that the CLL tumoral environment impacts healthy B cells as it does for T cells, NK cells and monocytes. Our study reveals two types of anomalies in the pre-immune healthy B cell compartment of the patients: a profound attrition of naïve B cells and a strong reduction of a population of anergic self-reactive B cells. Altogether our results suggest that CLL is accompanied by defective B cell lymphopoiesis and breaches in the B cell tolerance checkpoints.

## Materials and methods

### Study populations

Blood samples from CLL patients were obtained from primary diagnosis and treatment-naïve CLL patients (n = 11) under informed consent from the Hematology Department of the Centre Léon Berard, Lyon, France. The procedure was approved by health authorities according to national ethical recommendations and conducted with respect of the Declaration of Helsinki.

Age-matched healthy donors were recruited *via* the french blood bank (Etablissement Français du Sang) in Lyon, France according to standardized procedures. These blood samples were also obtained under informed consent.

### Sample processing

Peripheral blood mononuclear cells (PBMC) were isolated from 35 to 50 mL of blood freshly drawn on heparin tubes with Lympholyte-H Cell separation Medium (Cedarlane, Burlington, Canada). Freshly isolated PBMC were either immediately processed for CyTOF staining or cryopreserved at -150°C.

### Flow cytometry

A panel of 7 fluorochrome-conjugated mAbs directed against ROR-1, CD19, CD20, CD5, Ig kappa, Ig lambda and CD79b (**Supplemental Table 1**) was used to discriminate healthy from malignant B cells in the peripheral blood of CLL patients. Cell samples were run on a FACS CANTO II cytometer (BD Biosciences) and data were analyzed with the BD FACS Diva FlowJo softwares (v10.8.1, BD Biosciences). For IGHV analysis B cells were sorted on a FACS ARIA II (BD Biosciences) after staining with ROR-1, CD5, CD79, CD19 and CD20.

### Clonality analysis

Genomic DNA was extracted from sorted B cells using QIAamp DNA mini kit (Thermo Fisher Scientific). Analysis of clonality was performed according to the Biomed-2 protocols (12) by amplifying immunoglobulin (IG) heavy chain variable (VH) region gene rearrangements followed by capillary electrophoresis of the polymerase chain reaction (PCR) products for complementarity determining region 3 (CDR3) spectratyping. Clonal leukemic rearrangements were further characterized by direct Sanger sequencing as previously described (13).

### Analysis of *TP53* mutation status

Sanger sequencing (SS) was used for the mutational analysis of *TP53.* This test was performed at diagnosis for each patient as part of the study of molecular prognostic factors of the disease. The SS analysis of the entire coding region of *TP53* (exon 2-11) was conducted using the Applied Biosystems 3730 DNA analyzer (Applied Biosystems, Foster City, CA, USA).

### CyTOF antibody labeling and cell staining

Sample preparation. Cryopreserved PBMC were thawed, resuspended in phosphate buffer saline (PBS) to a concentration of 1-3x10^6^ cells/mL and incubated 10 min with FcR Block Reagent (Miltenyi Biotec) prior to Ab staining.

Staining procedure. Purified antibodies were either purchased pre-conjugated from the manufacturer (Fluidigm, San Francisco, CA) or conjugated in-house with the appropriate metal isotope using MaxPar Metal labeling kits (Fluidigm) according to manufacturer’s instructions. For staining, 1x10^6^ cells were washed in Maxpar cell staining buffer (Fluidigm) and stained in 100 μL Maxpar cell staining buffer (Fluidigm) containing the cocktail of 24 Abs listed in **Supplemental Table 1**. Stained cells were then incubated for 10 min in 1.6% paraformaldehyde (Sigma). DNA staining was performed by overnight incubation in 2 mL of 125 nM Cell-ID Iridium intercalator solution (Fluidigm) at 4°C. Cells were then washed, pelleted, and kept at 4°C until acquisition.

Data Acquisition. Samples were analyzed on a CyTOF2 mass cytometer upgraded to Helios (Fluidigm). Cells were resuspended in EQ™ Four Element Calibration Beads (Fluidigm) diluted to 0.5X in Maxpar ultra-pure water (Fluidigm) and filtered twice through a 50 µm nylon mesh to reach an acquisition rate of 200-500 events per second.

### Data processing and analysis pipeline

Pre-processing and preliminary manual gating. Signal normalization, concatenation and conversion into .fcs files were performed using the CyTOF2 Software (Fluidigm). Fcs files were imported into FlowJo and arcsinh-transformed (cofactor = 5). Gaussian parameters of the Helios system were used for doublet exclusion, then ^191^Ir^+193^Ir^+^ single events were manually isolated, and debris (CD45^-^ events) and calibration beads (^140^Ce^+^ events) were excluded (14). The CD45^+^ or gated CD19^+^ healthy B cell events were exported into separated files and uploaded in R or FlowJo for downstream analysis.

Dimensionality reduction was performed with the t-SNE (t-Distribution Stochastic Neighbor Embedding) (15) algorithm for mononuclear cells and with the UMAP (Uniform Manifold Approximation and Projection) (16) algorithm for B cells. In the latter case, the markers considered for UMAP were: CD5, CD10, CD11c, CD19, CD20, CD21, CD23, CD24, CD27, CD38, CD45, CD79b, IgM, IgD and HLA DR. t-SNE maps were created with the FlowJo software. 2D UMAP embeddings were generated thanks to the umap function of the uwot R package. Default parameter settings were adopted except the number of neighboring points which was set to 20.

FlowSOM metaclustering. 2D FlowSOM analyses were realized thanks to the FlowSOM Bioconductor’s package (using 10x10 grid size, on transformed and unscaled data) with a fixed pre-defined number of clusters and specific markers to be considered. A dataset composed by merging same sized subsamples of cells of each one of the healthy samples was considered to build the flowSOM models. After models building, in order to have cluster assignation for all cells of both healthy and CLL samples, FlowSOM models were then applied to each sample’s full data.

### Statistical analysis

Tests for differential abundance of cell populations among subgroups are based on the Wilcoxon rank sum test (equivalent to the Mann-Whitney test). Spearman’s rho statistic was used to test for possible association between clinical parameters and clusters frequencies. Spearman’s rho statistic estimates a rank-based measure of association.

## Results

### Discrimination of non-malignant and leukemic B cells by conventional flow cytometry

**Figure 1:**
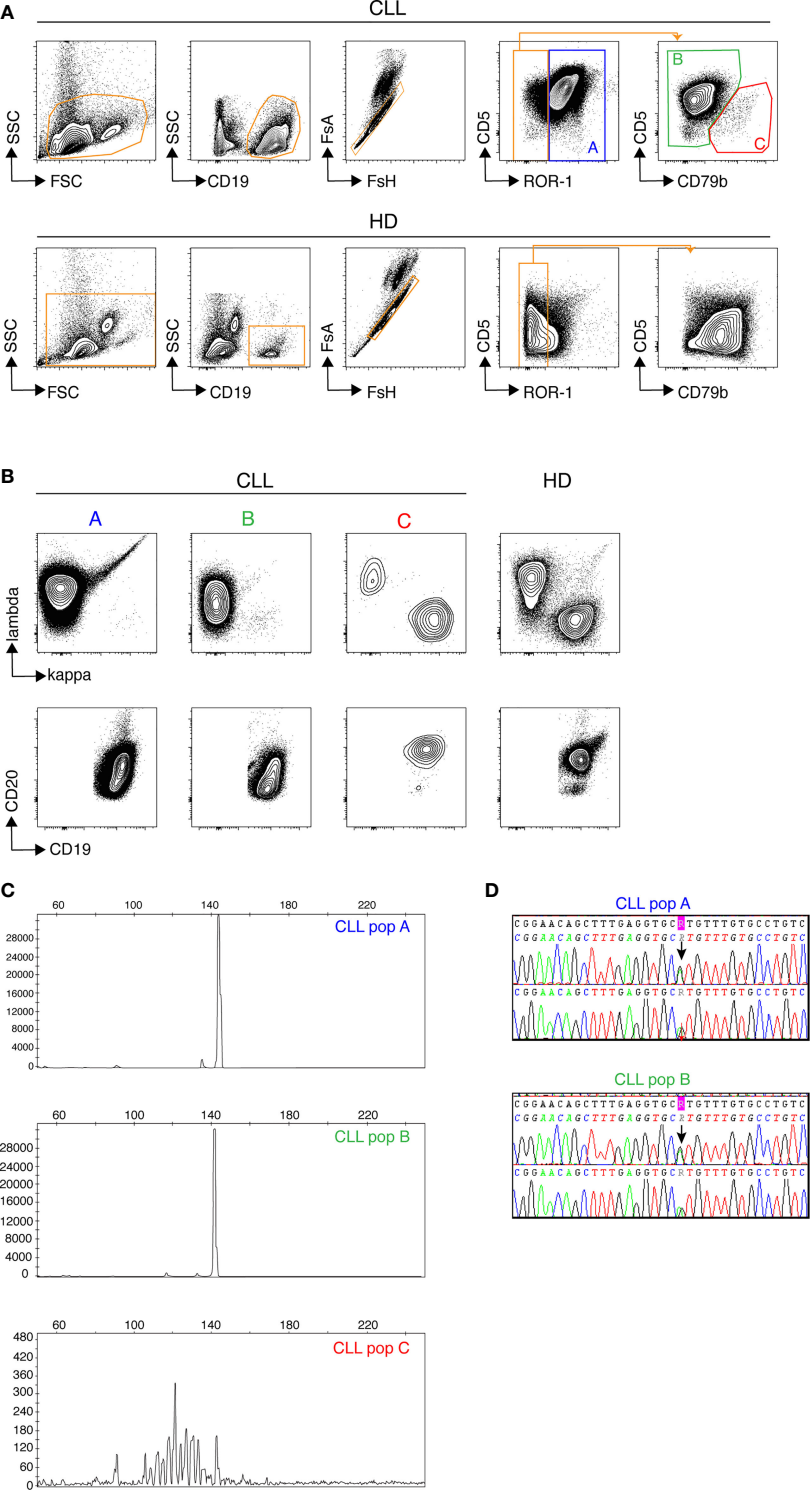
Discrimination of non-malignant and tumoral B cells from CLL samples by flow cytometry. Samples from healthy donors (HD) and CLL patients were stained with a cocktail of 7 fluorochrome-conjugated Abs recognizing, CD5, CD19, CD20, CD79b, ROR-1, Ig κ and Ig λ light chains. **(A)** Gatings from left to right: morphology, B cells, single cells, CD5/ROR-1 expression and CD5/CD79b expression within the ROR-1^lo/-^ gate. Results from one CLL (top row) and one representative HD (bottom row) sample are shown. **(B)** Expression of Igκ/Igλ (top row) and CD20/CD19 (bottom row) by the **(A–C)** subsets. One representative CLL patient (3 contour plots on the left) and one representative HD (4^th^ contour plots on the right). **(C)** Assessment of clonality by analysis of HCDR3 length in sorted CLL subsets **(A–C)** delineated in **(A)**, one representative result chosen among three. **(D)** Results of *TP53* mutational analysis in populations **(A, B)** performed by Sanger sequencing showing the presence of a common *TP53* mutation in populations **(A, B)** from the same patient.

We first used conventional flow cytometry to test whether a panel of 7 markers that are widely used either to define CLL cells (ROR-1, CD5) or to assess measurable residual disease (CD19, CD20, CD79b, kappa and lambda Ig light chains) (17) would allow for unambiguous identification of leukemic B cells. ROR-1 is a receptor tyrosine kinase-like orphan receptor documented to be expressed by CLL cells and by a subset of normal B cell precursors in the human bone marrow (18). As shown in **Figure 1A** (upper row), CD19^+^ B cells from CLL patients contained a prominent ROR-1^+^CD5^hi^ subset (population A). By contrast, ROR-1 was expressed only on a minor fraction of healthy donors (HD) B cells and at low density (**Figure 1A**, lower row). To analyze the identity of ROR-1^lo/-^ B cells in CLL samples, we relied on the expression intensities of CD5 and CD79b which are low and high, respectively, on healthy B cells (**Figure 1A**, lower row). Unexpectedly, patients ROR-1^lo/-^ B cells included two subtypes defined as CD5^hi^CD79b^lo^ (population B) and CD5^lo^CD79b^hi^ (population C). As shown in **Figure 1B**, gated populations A and B were both CD20^lo^ and expressed a single Ig light chain isotype (Igλ for the patient shown) as expected from leukemic B cells (19). By contrast, cells from population C were CD19^hi^CD20^hi^ and segregated into either κ- or λ-bearing types. Thus, despite its low to negative expression of ROR-1, population B shared the phenotypical attributes of the malignant clone, while population C presented the phenotypical features of non-malignant B cells. Populations A, B and C were next sorted from PBMC of CLL patients (n= 3) based on their pattern of expression of ROR-1, CD5, CD20, CD19 and CD79b and their IGHV clonality was assessed by HCDR3 length spectra-typing. As illustrated by **Figure 1C**, CLL population C (ROR-1^lo/-^CD5^lo/-^CD79b^hi^) displayed a polyclonal CDR3 length spectrum while population B (ROR-1^lo/-^CD5^hi^CD79b^lo^) displayed the same monoclonal CDR3 peak size as the ROR-1^+^ leukemic clone (population A). Sequencing of the VH regions of populations A and B sorted from three distinct CLL specimens revealed that they possessed identical sequences including in their CDR3 and thus belonged to the same leukemic clone (**Table 1**). Moreover, population B sorted from a CLL patient with a molecular mutation of the *TP53* gene, presented the same mutational hallmark as that found in the ROR-1^+^ population A (**Figure 1D**). Together, these findings indicate that the malignant B cell pool although predominantly ROR-1^+^ also includes ROR-1^lo/-^ B cells. Thus, the sole expression of ROR-1 is not sufficient to stringently exclude tumoral cells from the peripheral B cell compartment of CLL patients.

**Table 1:** IGHV clonality assessment of B-CLL healthy B cell subsets by CDR3 region sequencing.

Population	IGHV	Identity to germline (%)	IGHD	IGHJ	Reading frame	CDR3 length (AA)	CDR3 sequence (AA)
Patient #1							
A	V3-33*01	100	D3-22*01	J4*02	IF	19	CARAAKRVPYYYDSSGPFDYW
B	V3-33*01	100	D3-22*01	J4*02	IF	19	CARAAKRVPYYYDSSGPFDYW
Patient #2							
A	V1-2*02	92.01	D2-2*01	J6*02	OF	ND	CAWLPYGLG#MDVW
B	V1-2*02	92.01	D2-2*01	J6*02	OF	ND	CAWLPYGLG#MDVW
Patient #3							
A	V3-30*03	100	D3-3*01	J5*02	IF	22	CAKDYTITTYYDFWSGYYPNWFDPW
B	V3-30*03	100	D3-3*01	J5*02	IF	22	CAKDYTITTYYDFWSGYYPNWFDPW

AA, amino-acid.

ND, not determined.

### Unsupervised discrimination of healthy hematopoietic cells and leukemic B cells

**Figure 2:**
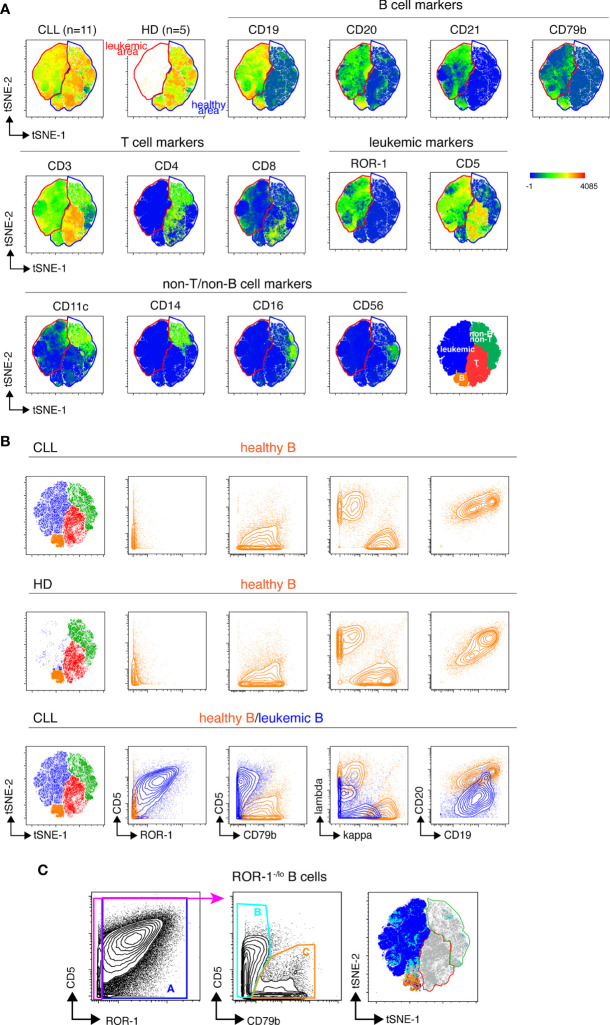
Unsupervised definition of the non-malignant and leukemic B cell pools in CLL samples by mass cytometry. **(A)** Top row: CLL and HD t-SNE maps obtained after analysis of the PBMC from 11 CLL and 5 HD samples stained with the 24-marker panel (concatenated file including data from both the HD and CLL samples). The healthy B cell area, common to CLL and HD is gated in blue, the leukemic B cell area that appears only in the CLL samples is gated in red. Middle and lower row: marker expression shown as heat plots on the t-SNE map of concatenated CLL files. Bottom right: representation of the areas occupied on the t-SNE map by leukemic cells and healthy T, B and non-T non-B cells colored by cell-type assignments. **(B)** Validation of the leukemic and healthy areas. Expression of CD5, ROR-1, CD79b, Ig light chains, CD19 and CD20 by healthy B cells from CLL patients (upper row) or from HD (middle row) manually-gated on the t-SNE maps of merged HD or-CLL samples. Lower row, overlay of non-malignant and leukemic B cells from the merged CLL samples. Note that Igκ and Igλ light chains are apparently co-expressed on leukemic B cells because the analysis is conducted on 11 CLL leukemic clones that individually express distinct Ig light chain isotypes. **(C)** Distribution of ROR-1^+^ and ROR-1^-/lo^ leukemic B cells within the leukemic and healthy B cell areas of the merged CLL t-SNE map. Left: ROR-1^+^ (population A) and ROR-1^-/lo^ B cells are manually gated on a ROR-1/CD5 bi-parameter plot. Middle: contour plots showing the pattern of CD79b and CD5 expression by ROR-1^-/lo^ B cells and the gating of CD79b^lo^/CD5^+/-^ B cells (pop B, pale blue) and CD79b^hi^/CD5^lo^ B cells (pop C, orange). Right: manually-gated populations **(A–C)** are overlayed onto the merged CLL t-SNE map.

We next designed a B cell-centered panel of 24 monoclonal Abs conjugated to rare-earth heavy-metal isotopes to conduct in-depth phenotypical analysis of the non-malignant B cell pool of treatment-naïve CLL patients using single-cell mass cytometry (cyTOF). Our first goal was to determine whether unsupervised analysis of the stainings conducted with this panel could reliably permit to discriminate healthy from leukemic B cells in CLL samples. To do so, stainings were performed on PBMCs isolated from 11 CLL patients (**Supplemental Table 2**) and from 5 age-matched healthy donors (HD). The mass cytometry data from the HD and CLL specimens were then merged and submitted to dimensionality reduction using the t-SNE algorithm (15). As shown in **Figure 2A**, two phenotypic regions were clearly separated on the t-SNE map of the CLL specimens. One of them being occupied in both leukemic and HD samples presumably corresponded to healthy hematopoietic cells. The other being occupied only in the leukemic sample thus contained cells with a “different-from-normal” phenotype and was considered as the “leukemic area”. The expression profile of CD3, CD4, CD5, CD8, CD11c, CD14, CD16, CD19, CD20, CD21, CD56, CD79b and ROR-1 allowed for identification of four sub-regions of the t-SNE map corresponding to: leukemic B cells, healthy B cells, T cells and non-T/non-B cells, respectively (bottom right t-SNE plot). To validate this leukemic B cell exclusion strategy, cells of the leukemic and non-malignant B cell compartments were manually gated on the t-SNE map and examined for their pattern of expression of the 7 markers described previously. As shown in **Figure 2B**, cells falling within the healthy B cell area of the CLL t-SNE map upper panel) were ROR-1^-^CD5^lo^CD79b^hi^Igκ/λ^hi^ CD19^hi^CD20^hi^, i. e. phenotypically similar to HD B cells (middle panel). We next compared leukemic and healthy B cells gated on the t-SNE map of CLL patients (**Figure 2B**, lower panel). As opposed to non-malignant B cells (in orange), cells that fell within the leukemic area of the CLL samples (in blue) were ROR-1^hi^CD5^hi^CD79b^lo^Igκ/λ^lo^CD19^lo^CD20^lo^. To examine whether ROR-1^lo/-^ leukemic B cells also segregated within the leukemic area of the t-SNE map, ROR-1^lo/-^ B cells manually gated on the CD5/ROR-1 bi-parameter plot of merged CLL specimens were further separated into CD79b^lo^/CD5^hi^ (population B, leukemic) and CD79b^hi^/CD5^lo^ (population C, healthy) cells. Projection of these two B cell subtypes onto the CLL t-SNE map (using differently colored dots for each subset) showed that population B (light blue) fell within the leukemic area, while population C (orange) fell within the non-leukemic area (**Figure 2C**). Altogether these data indicate that unsupervised analysis of stainings performed with the 24-marker panel allows stringent exclusion of leukemic B cells, including those that are ROR-1^lo/-^.

### Dissection of the non-malignant peripheral B cell pool of CLL patients.

**Figure 3:**
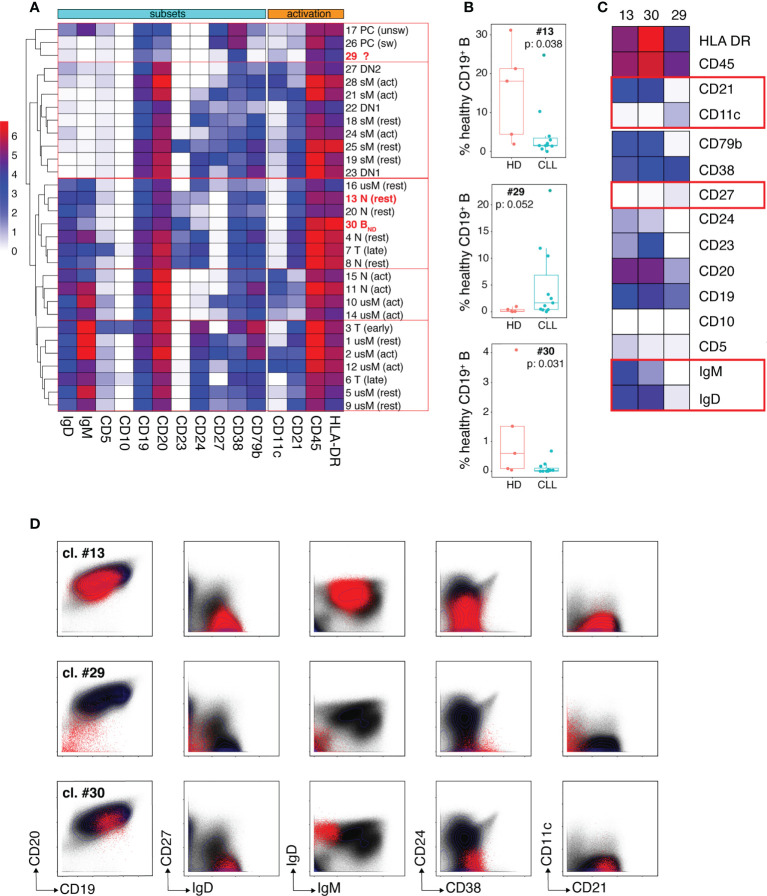
Dissection of the non-malignant peripheral B cell compartment of CLL patients. **(A)** Heatmap showing median intensity of the indicated markers for the 30 healthy B cell clusters individualized by the FlowSOM algorithm and their clustering results as performed by the hclust software. The phenotypical criteria used for manual annotation of the clusters are described in the results section. T: transitional B cells, N: naïve B cells, usM, sM, DN1 and DN2 stand for unswitched conventional mBCs, switched conventional mBCs and double negative mBCs type 1 and 2, respectively. PCs: plasma cells. The 3 clusters which frequency modulation in CLL patients was statistically significant are identified in bold red. **(B)** Box and whisker plots of the frequencies of cells (expressed in % of the whole healthy B cell population) for the three clusters (13, 29 and 30) that showed disease-associated modulation. **(C)** Heatmap showing median intensity of the indicated markers for clusters 13, 29 and 30. The red boxes identify markers which are determinant for cluster-to-B cell subtype assignment. **(D)** Biaxial plots showing expression of the indicated markers by cells from clusters 13, 29 and 30 visualized as red dots overlayed onto the expression profile of the markers on the entire healthy B cell population (black dots). In **(B)** box and whisker plots are shown with the median, interquartile range (IQR, a box with lower and upper bonds representing 25th and 75th percentiles, respectively) and 1.5 times the IQR (whiskers).

We undertook an unsupervised dissection of the healthy B cell pool of CLL patients by performing a clustering analysis on the merged mass cytometry data (HD + CLL samples) corresponding to gated healthy B cells, using the FlowSOM algorithm (20). Fourteen markers were considered for clustering: CD5, CD10, CD11c, CD19, CD20, CD21, CD23, CD24, CD27, CD38, CD79b, HLA DR, IgM and IgD. The parameters of the FlowSOM analysis were set in order to discriminate 30 clusters. A heatmap of median marker expression was generated to visualize the phenotypical profile of each cluster and hierarchical clustering (hclust algorithm) was applied to group phenotypically close clusters (**Figure 3A**). All 30 clusters were also manually assigned to known B cell types according to their phenotypical signature, based on published literature (**Supplemental Table 3**). Resting (CD21^hi^CD11c^-^) and activated (CD21^lo^CD11c^+^) B cells were discriminated according to their pattern of CD21 and CD11c expression as described by Lau and colleagues (21). Transitional B cells (T) were defined as IgD^+^CD27^-^CD24^hi^CD38^hi^ cells (22, 23). Early (i. e. T1/T2) and late (i. e. T3) transitional B cells were identified as CD10^+^ and CD10^lo^ cells, respectively. Naïve B cells (N) were defined as IgD^+^CD27^-^CD24^lo^CD38^lo^ cells. They were subdivided into: i) resting N, ii) activated N (or ABC/Age-associated B Cells (24)) and iii) anergized autoreactive N, a subset referred to in the literature as B_ND_ cells (25, 26). Unswitched (usM), conventional switched (sM) and double negative (DN) memory B cells (mBCs) were defined as IgD^+^CD27^+^, IgD^-^CD27^+^ and IgD^-^CD27^-^ cells, respectively. DN cells were further subdivided into DN1 (CD21^hi^CD11c^lo^) and DN2 (CD21^lo^CD11c^+^) subtypes as described by the group of I. Sanz (27, 28). Plasma cells (PCs) were defined as CD19^+^CD20^lo^CD27^hi^CD38^hi^ cells. Unswitched PCs were distinguished from their switched counterparts by the remanence of membrane IgM on the former subtype (29).

Only clusters for which disease-dependent frequency modulation was statistically significant were considered further. Three clusters fell into this category: clusters 13 and 30 which were under-represented in CLL patients and cluster 29 which was expanded in the patients (**Figure 3B**). The phenotypical traits of these three clusters are illustrated by the heat-map of median marker expression in **Figure 3C** and by a series of bi-parameter plots shown in **Figure 3D**. Cluster 13 was assigned to resting naïve B cells because it co-expresses IgM and IgD, lacks CD27 (which excludes an usM identity), expresses CD21 and lacks CD11c (which rules out activation). The median frequency of cluster 13 was reduced more than 10-fold in the patients (1.58% *vs* 18% in HD). The phenotypical characteristics of cluster 30 (IgD^+^CD10^-^CD24^-^CD27^-^) excluded a transitional, sM, usM and DN identity. Its very low IgM expression was highly evocative of the phenotype of B_ND_ cells (naïve IgD^+^IgM^-^) (25, 26). B_ND_ cells are described as B cells that have survived the B cell tolerance checkpoints and are maintained in the periphery but with reduced functional capacity. Cluster 30 that accounts for 0.6% of B cells (median value) in HD, constituted only 0.029% of the peripheral non-malignant B cell pool of CLL patients, which represents a more than 20-fold reduction.

Cluster 29 was virtually undetectable in HD but constituted a very prominent B cell subset for some of the patients. Cells from cluster 29 segregated with the PC cluster group according to hierarchical clustering because of their low expression of CD20 which constitutes one of the distinctive phenotypical traits of PCs. However, they lacked the canonical features of PCs, i. e. high density of CD27 and CD38 expression (30, 31). They also displayed similarities with DN2 memory B cells (the CD21^lo^CD11c^+^ module) described by the group of I. Sanz as extra-follicular Ab-secreting cell precursors (27, 28, 32) but their low levels of CD19 and CD20 expression is unprecedented for a memory B cell subset. This unconventional marker combination suggests that these cells may represent a non-classical B cell population at an intermediate differentiation stage between DN2 and PCs.

### Correlations with clinical and biological features of the disease

**Figure 4:**
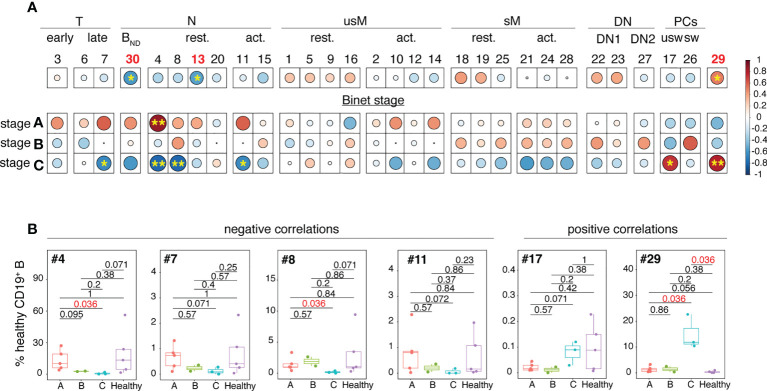
Analysis of the inter-relationships between non-malignant B cell cluster frequency and Binet stages. **(A)** Clusters have been organized and grouped according to the B cell developmental stage they belong to. The color of the circles corresponds to the correlation (red for positive, blue for negative) and the stars and size of the circles indicate the degree of significance of the correlation (*p < 0.05, **p < 0.01). **(B)** Box and whisker plots illustrating the frequencies of the clusters which abundance is correlated with Binet stage C in stage A, B and C patients. Statistical significancy was estimated with the Mann-Whitney U test. The p values are indicated on the graphs. P values of <0.05 were considered as statistically significant and are identified in red.

Spearman’s correlation coefficients were next used to test the possible association between clinical parameters of the disease and frequencies of non-malignant B cell clusters. As shown in **Figure 4A**, four clusters negatively correlated with Binet stage C. They belonged either to the transitional (cluster #7) or naïve (clusters # 4, 8 and 11) B cell developmental stages. The frequency disparities between Binet stage A, B and C for these clusters did not reach statistical significance (**Figure 4B**) but for three of them (#4, #7 and #11) there was a tendency towards a gradual reduction in abundance from stage A to stage C.

Conversely, positive correlations between cluster frequency and Binet stage C were restricted to unswitched PCs (cluster #17) and to the atypical PC-related cluster 29. Association between elevated frequency of cluster 29 and Binet stage C was strong as the difference between stage C patients and HD reached statistical significance (p = 0.036).

Altogether these results indicate that the non-malignant B cell pool of CLL patients is characterized by attrition of naïve B cells including an anergized self-reactive subset. Inverse correlations between Binet stage C and abundance of naïve B cell clusters suggest that reduced frequency of naïve B cells may be associated with disease severity.

## Discussion

The unsupervised analytical approach used here has allowed us to reveal subtle variations in composition of the patients’ healthy B cell pool that might have been missed by supervised analysis. More specifically, our data reveal attrition of two populations in the pre-immune B cell compartment of CLL patients: naïve B cells and a subset of anergic self-reactive naïve B cells designated as B_ND_. Our findings are in agreement with two studies which documented that production and release of immature and naïve B cells in the periphery is reduced in monoclonal B cell lymphocytosis and CLL (33, 34). Yet, the strong reduction of the abundance of B_ND_ cells had not yet been documented and suggests that attrition of the naïve B cell compartment is not the mere consequence of a competition between healthy B cell precursors and invading leukemic B cells. B_ND_ cells are IgM^lo/-^ naïve B cells that express an autoreactive BCR and have been functionally censored after recognition of self Ag during early development (25, 26). It is also documented that the B_ND_ pool serves as a reservoir for autoreactive B cells in several autoimmune diseases (35). At face value, it may seem counterintuitive to associate decreased abundance of B_ND_ cells with an increased risk of autoreactivity. However, Smith and colleagues (36) have reported that insulin-binding B cells are found in the B_ND_ compartment of healthy subjects but are no longer detected in the B_ND_ pool of patients who have developed an active diabetes and anti-islet Abs. They go on to show that that these cells are absent from the anergized naïve compartment because they have escaped from their tolerant state to differentiate into auto-Ab producing cells. In this context, attrition of the B_ND_ subset in CLL patients could be seen as an indication that immune tolerance checkpoints have been overridden. In our study, only two patients showed signs of autoimmune manifestations (hemolytic anemia) but the frequency of their B_ND_ cells was not significantly lower than that of the patients that failed to develop autoimmunity. Although this finding should be interpreted with caution due to the small numbers of patients considered, it tends to exclude the possibility to exploit B_ND_ cells as a predictive tool for future onset of autoimmunity in CLL patients. We rather propose that contraction of the B_ND_ pool may contribute to a general predisposition of CLL patients to autoimmunity. Because the strongest reduction of naïve B cells coincides with Binet stage C, the degree of attrition of this population may be a marker of disease severity. Abundance of cluster 29 is strongly increased in CLL specimens especially in Binet stage C patients, i. e. those with a rapidly progressing disease. This suggests a very strong association between emergence of this atypical B cell subtype and less favorable evolution of the disease. Unfortunately, this cell cluster is unclassifiable so far, at least with the Ab panel used in our study.

Methodologically speaking, it should be emphasized that the groups of phenotypically-related B cell subtypes created by the hierarchical clustering algorithm do not perfectly overlap with the categories defined by manual assignment of B cell subtype identity. Thus, automated hierarchical clustering proved to be at least partially inaccurate for cell type assignment as compared to the cluster-then-annotate approach used here. On the other side, manual annotation of cell clusters is possibly biased because it relies on user expertise. A large consensus needs to be found on the precise phenotypical attributes of the multiple subsets constituting the peripheral B cell pool in order to create an expert-curated reference data base.

In conclusion, the results of our pilot study suggest that CLL creates an altered milieu providing the conditions for reduced output of naïve B cells and perturbation of the B cell tolerance mechanisms. It remains to determine what are the respective contributions of malignant cells and of surrounding non-tumor cells engaged in the disease process to the B cell anomalies reported here. Our work provides elements to guide future analysis of larger groups of CLL patients possibly stratified on the basis of clinical criteria such as autoimmune manifestations which may be linked to some of the developmental anomalies we report here.

## Data availability statement

The raw data supporting the conclusions of this article will be made available by the authors, without undue reservation.

## Ethics statement

The studies involving human participants were reviewed and approved by Hematology Department of the Centre Léon Berard, Lyon, France. The patients/participants provided their written informed consent to participate in this study.

## Author contributions

TA, PM, SD, VM, and HS performed the experimental studies. TA designed the Ab panels, ran the mass cytometer, carried out computational studies and bioinformatics analysis and contributed to edition of the manuscript. P-EJ carried out computational studies and bioinformatics analysis and contributed to edition of the manuscript. LB contributed to the recruitment of patients. A-SM contributed to the recruitment of patients, to the design of the study and edition of the manuscript. FD performed experimental studies and contributed to analysis of the data and edition of the manuscript. TD designed the study, analyzed the data, supervised the work and wrote the manuscript. All the authors have approved the content of this paper.

## Funding

This work was supported by the National and Regional Committees of la Ligue Contre le Cancer and the Janssen Laboratory.

## Acknowledgments

The authors would like to thank all the patients who participated in the study as well as medical professionals involved in blood sampling at the Centre Léon Bérard and Etablissement Français du sang (EFS, Lyon). We are grateful to Olivier Thaunat and Helena Paidassi for their expert proofreading of the manuscript and for their valuable comments and suggestions.

## Conflict of interest

Author P-EJ was employed by company AltraBio SAS. Author HS was employed by company France Biotech.

The remaining authors declare that the research was conducted in the absence of any commercial or financial relationships that could be construed as a potential conflict of interest.

## Publisher’s note

All claims expressed in this article are solely those of the authors and do not necessarily represent those of their affiliated organizations, or those of the publisher, the editors and the reviewers. Any product that may be evaluated in this article, or claim that may be made by its manufacturer, is not guaranteed or endorsed by the publisher.
