# Supplementary material for: Mass cytometry analysis reveals attrition of naïve and anergized self-reactive non-malignant B cells in chronic lymphocytic leukemia patients
Source: Front Oncol. 2022 Oct 31;12:1020740. doi: 10.3389/fonc.2022.1020740 (PMC9661965; doi:10.3389/fonc.2022.1020740)
Supplement: Supplementary file 1 [file Table_1.docx]

**Supplemental Table 1. Staining panels for flow and mass cytometry.**

|  | **Mass cytometry** | | | **Flow cytometry** | |
| --- | --- | --- | --- | --- | --- |
| **Marker** | **Clone** | **Isotope** | **Source** | **Clone** | **Fluorochrome** |
| **CD3** | HIT3a | 159Tb | BD |  |  |
| **CD4** | RPA T4 | 153Eu | BD |  |  |
| **CD5** | UCTH2 | 158Gd | BD | L17F12 | PE Cy7 |
| **CD8** | RPA-T8 | 142Nd | BD |  |  |
| **CD10** | HI10a | 165Ho | BD |  |  |
| **CD11c** | B Ly6 | 171Yb | BD |  |  |
| **CD14** | M5E2 | 155Gd | BD |  |  |
| **CD16** | 3G8 | 148Nd | BD |  |  |
| **CD19** | HIB19 | 166Er | BD | HiB19 | BV 421 |
| **CD20** | 2H7 | 152Sm | BD | L27 | V 500 |
| **CD21** | B Ly4 | 168Er | BD |  |  |
| **CD23** | M L233 | 175Lu | BD |  |  |
| **CD24** | ML5 | 164Dy | BD |  |  |
| **CD27** | M T271 | 176Yb | BD |  |  |
| **CD38** | HB 7 | 144Nd | ebiosciences |  |  |
| **CD45** | HI30 | 173Yb | fluidigm |  |  |
| **CD56** | B159 | 146Nd | BD |  |  |
| **CD79b** | 3A2 2E7 | 170Er | BD | SN8 | PE |
| **Ig kappa** | G20 193 | 147Sm | BD | G20 193 | APC H7 |
| **Ig lambda** | MHL 38 | 156Gd | BD | 1-155-2 | FITC |
| **IgM** | G20 127 | 169Tm | BD |  |  |
| **IgD** | IA6 2 | 141Pr | BD |  |  |
| **HLA-DR** | L203 | 143Nd | BD |  |  |
| **ROR-1** | 4A5 | 161Dy | BD | poly. | AF 647 |

All mAbs used for flow cytometry were purchased from Becton-Dickinson Biosciences, France, except the AF 647-conjugated goat-anti-human ROR-1 polyclonal Ab that was purchased from R and D Systems.
