# Supplementary material for: Mass cytometry analysis reveals attrition of naïve and anergized self-reactive non-malignant B cells in chronic lymphocytic leukemia patients
Source: Front Oncol. 2022 Oct 31;12:1020740. doi: 10.3389/fonc.2022.1020740 (PMC9661965; doi:10.3389/fonc.2022.1020740)
Supplement: Supplementary file 2 [file Table_2.docx]

**Supplemental Table 2. Clinical data for the CLL patient cohort.**

| Patient  ID | Gender | Age  (years) | Binet  stage | *TP53*  status | Cytogenetic  abnormalities | IGHV mut. status | Hypo  gamma | Hemolytic  Anemia |
| --- | --- | --- | --- | --- | --- | --- | --- | --- |
| CLB#3 | Male | 49 | A | ND | del 13q | ND | no | no |
| CLB#4 | Male | 72 | C | mutated | tri 12 | unmutated | yes | no |
| CLB#5 | Male | 61 | A | ND | del 13 q | ND | yes | no |
| CLB#6 | Male | 74 | A | ND | del 13q | mutated | no | no |
| CLB#7 | Male | 67 | A | unmutated | none | mutated | no | yes |
| CLB#8 | Male | 79 | C | unmutated | del17p | ND | no | no |
| CLB#9 | Female | 66 | B | ND | del 13 p | ND | no | no |
| CLB#10 | Male | 72 | B | unmutated | del 13 q | mutated | yes | no |
| CLB#12 | Female | 76 | C | unmutated | none | mutated | yes | yes |
| CLB#15 | Male | 69 | A | unmutated | del 13 q | mutated | yes | no |
| CLB#16 | Female | 69 | A | ND | ND | ND | ND | ND |

None of the patients above had received any specific treatment at the time of blood sample collection. ND : not determined.

Hypogamma: hypogammaglobulinemia.
