# Supplementary material for: Mass cytometry analysis reveals attrition of naïve and anergized self-reactive non-malignant B cells in chronic lymphocytic leukemia patients
Source: Front Oncol. 2022 Oct 31;12:1020740. doi: 10.3389/fonc.2022.1020740 (PMC9661965; doi:10.3389/fonc.2022.1020740)
Supplement: Supplementary file 3 [file Table_3.docx]

**Supplemental Table 3. Phenotypical definition of circulating human B cell subsets.**

| **B cell population** | **Subtypes** | **Acronym** | **Markers** |
| --- | --- | --- | --- |
| Transitional | Early | T | IgD^+^/IgM^+^/CD10^+^/CD11c^-^/CD20^+^/CD21^-^/CD23^-^/CD24^hi^/CD27^-^/CD38^hi^ |
|  | Late |  | IgD^+^/IgM^lo^/CD10^lo^/CD11c^-^/CD20^+^/CD21^+^/CD23^-/+^/CD24^hi^/CD27^-^/CD38^hi^ |
| Naïve | Resting | N rest | IgD^+^/IgM^+^/CD10^-^/CD11c^-^/CD20^+^/CD21^+^/CD23^+^/CD24^+^/CD27^-^/CD38^lo^ |
|  | Activated | N act/ABC | IgD^+^/IgM^+^/CD10^-^/CD11c^+^/CD20^+^/CD21^lo^/CD23^-^/CD24^-^/CD27^-^/CD38^lo^ |
|  | Anergized | B_ND_ | IgD^+^/IgM^lo^/CD10^-^/CD11c^-^/CD20^+^/CD21^+^/CD23^hi^/CD24^lo^/CD27^-^/CD38^lo^ |
| Conventional mBCs | Unswitched resting | usM rest | IgD^+^/IgM^+^/CD10^-^/CD11c^-^/CD20^+^/CD21^+^/CD23^-^/CD24^+^/CD27^+^/CD38^lo^ |
|  | Unswitched activated | usM act | IgD^+^/IgM^+^/CD10^-^/CD11c^+^/CD20^+^/CD21^-^/CD23^-^/CD24^-^/CD27^+^/CD38^lo^ |
|  | Switched resting | sM rest | IgD^-^/IgM^-^/CD10^-^/CD11c^-^/CD20^+^/CD21^+^/CD23^+/-^/CD24^+^/CD27^+^/CD38^lo^ |
|  | Switched activated | sM act | IgD^-^/IgM^-^/CD10^-^/CD11c^+^/CD20^+^/CD21^-^/CD23^-^/CD24^-^/CD27^+^/CD38^lo^ |
| Atypical/DN mBCs |  | DN1 | IgD^-^/IgM^+/-^/CD10^-^/CD11c^-^/CD20^+^/CD21^+^/CD23^-^/CD24^+^/CD27^-^/CD38^lo^ |
|  |  | DN2 | IgD^-^/IgM^+/-^/CD10^-^/CD11c^+^/CD20^+^/CD21^-^/CD23^-^/CD24^-^/CD27^-^/CD38^lo^ |
| Plasma cells | Unswitched | usPC | IgD^-^/IgM^+^/CD10^-^/CD11c^-^/CD20^lo^/CD21^-^/CD23^-^/CD24^-^/CD27^hi^CD38^hi^ |
|  | Switched | sPC | IgD^-^/IgM^-^/CD10^-^/CD11c^-^/CD20^lo^/CD21^-^/CD23^-^/CD24^-^/CD27^hi^/CD38^hi^ |
