# Supplementary figures and images for: Mass cytometry analysis reveals attrition of naïve and anergized self-reactive non-malignant B cells in chronic lymphocytic leukemia patients
Source: Front Oncol. 2022 Oct 31;12:1020740. doi: 10.3389/fonc.2022.1020740 (PMC9661965; doi:10.3389/fonc.2022.1020740)

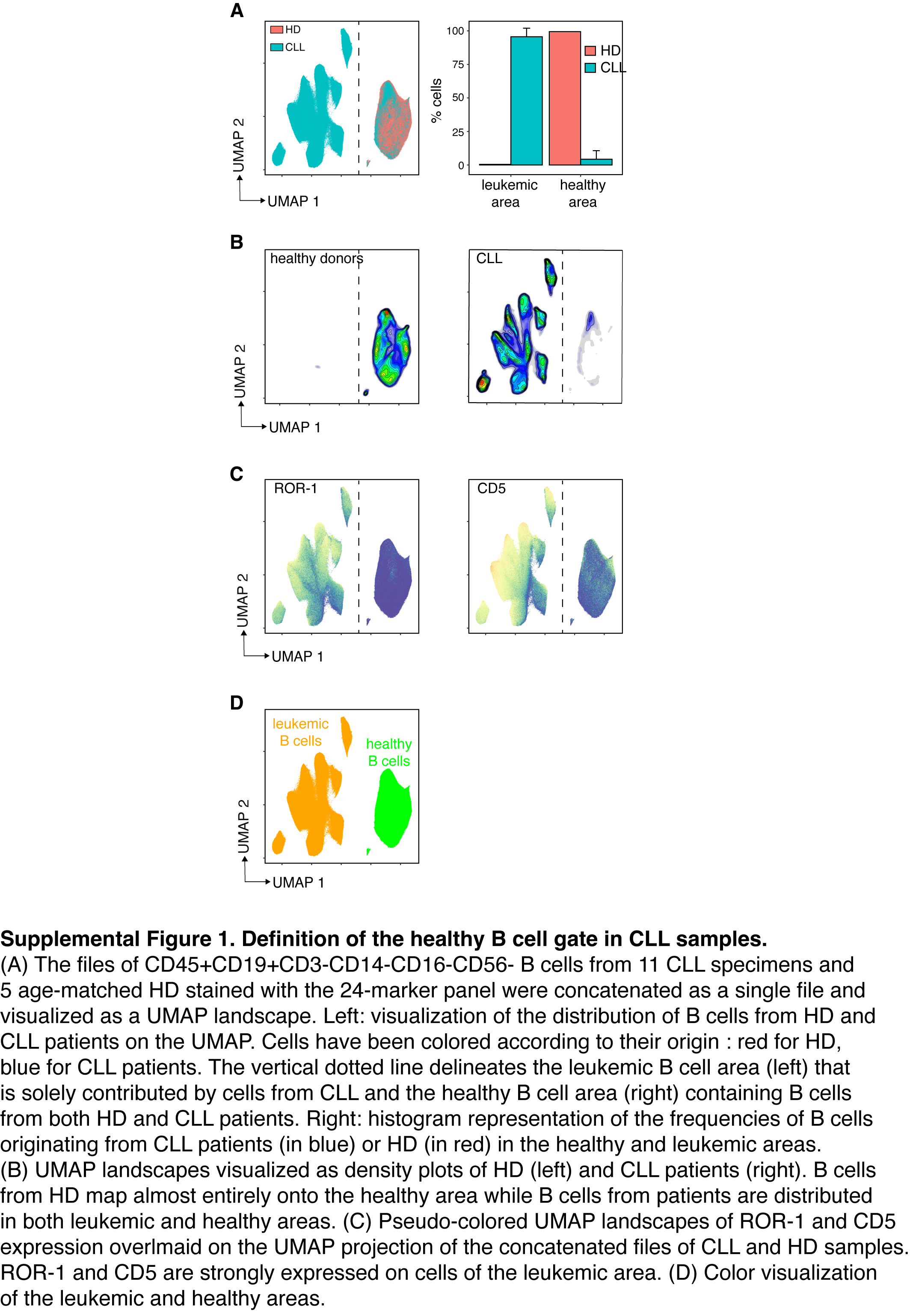

Supplement: Supplementary file 4 [file Image_1.tif]
